# Knowledge and practice of family planning among pregnant tribal women in Southern India: an observational study

**DOI:** 10.1186/s40834-023-00259-3

**Published:** 2024-01-12

**Authors:** Kiranmayee Muralidhar, Holly Nishimura, Kate Coursey, Karl Krupp, Poornima Jaykrishna, Vijaya Srinivas, Purnima Madhivanan

**Affiliations:** 1 Public Health Research Institute of India, Mysore, Karnataka India; 2 JSS Academy of Higher Education and Research, Mysore, Karnataka India; 3 Johns Hopkins Bloomberg School of Public Health, Baltimore, MD USA; 4 Department of Medicine, David Geffen School of Medicine, University of California, Los Angeles, CA USA; 5 Department of Health Promotion Sciences, Mel & Enid Zuckerman College of Public Health, University of Arizona, 1295 N. Martin Avenue, P.O. Box 245209, Tucson, Arizona 85724-5209 USA

**Keywords:** Contraception, Health literacy, Reproductive health, Tribal health, Women’s health, Family planning

## Abstract

**Background:**

There are over 700 Scheduled Tribes (ST) living in 30 Indian states. As with other indigenous groups across the world, Indian ST have some of the poorest infant and child health outcomes of any communities in India. A child born to an ST family is 19% more likely to die in the first month of life and has a 45 percent risk of dying in their first year compared with other Indian populations. Research suggests that early conception, high fertility, and low use of family planning methods are large contributors to these disparities.

**Methods:**

A cross sectional survey in *Kannada* was conducted among 303 pregnant tribal women in Mysore, India after obtaining informed consent. Univariate and multivariable analyses were carried out to determine the demographic and psychosocial factors associated with knowledge of contraceptive methods using Stata 14.0.

**Results:**

There was widespread knowledge about female sterilization, while only 39.3% of women reported hearing about one or more forms of temporary contraception, and 36.3% knew where to get them. The largest proportion of women had heard about copper-T (33.0%), followed by oral contraceptive pills (23.8%), condoms (11.9%), and injectables (4.6%). Only 2.7% of women reported ever using any form of temporary contraception. Results from the multivariable logistic regression indicated that knowledge of at least one form of temporary contraception was linked to higher age (adjusted odds ratio[AOR]: 1.09; 95% CI: 1.02, 1.17), greater number of years of marriage (AOR: 0.90; 95% CI: 0.85, 0.96), and last birth in a government facility (AOR: 3.67; 95% CI: 1.99, 6.82).

**Conclusions:**

The study revealed poor knowledge and utilization of temporary contraceptive methods among a tribal population in rural Mysore, India. Interventions aiming to increase knowledge of contraceptive options are important for birth spacing in this population and should target younger women and those without contact with government health facilities.

## Background

As with other indigenous groups across the world, Scheduled Tribes (ST) have some of the poorest infant and child health outcomes of any communities in India [1]. While the all-India neonatal mortality rate (NMR)—deaths in the first 28 days of life per thousand live births—was 20 per thousand in 2022, [2] NMR amongst ST ranged between 33 and 59 per thousand live births [3, 4]. Child deaths before the age of one were similarly almost 49% higher amongst ST than across India as a whole [2, 5]. Although the reasons for these disparities are multifactorial, [6] studies suggest that early conception, high fertility, and low use of family planning methods are large contributors [7, 8]. Contraception—defined as any intentional act to prevent pregnancy—is an essential component of family planning [9] and increased contraception usage has been associated with improved maternal and neonatal outcomes globally [10]. The Sustainable Development Goals call for universal availability of sexual and reproductive health services by 2030, with particular emphasis on satisfying family planning needs with modern contraceptive methods [11]. Given relatively poor neonatal outcomes amongst ST populations and other benefits of contraception such as reducing unintended pregnancies’ risks to the mother, it is critical to understand current contraception knowledge and practices to inform interventions that will promote contraception utilization in these groups. Our study was carried out among the *Jenu Kurubas*, also referred to as the *Kattu Nayaka*, a government recognized tribe occupying the border areas of Karnataka, Tamil Nadu, and Kerala in India.

Studies across India have noted relatively lower levels of contraception usage and greater unmet need for contraception amongst STs, especially compared to general caste groups [12, 13]. In particular, women from STs are less likely to utilize modern reversible forms of contraception, [14] which are widely considered to be the most reliable and effective means of preventing pregnancy. As per the latest National Family Health Survey, these disparities hold true at the state level—in Karnataka, only 65.0% of married women from STs are using some form of contraception, with 64.8% using a modern method, [15] which are lower percentages than other caste groups. However, given the significant heterogeneity amongst tribal groups across India, country and state-level data may not be reflective of contraception knowledge and usage within a specific scheduled tribe.

A review identified seven studies on contraceptive knowledge and use among Indian ST women from specific areas between 2010 and 2022 [16–22]. These studies—conducted mostly in Northeastern and Eastern India—highlight both the disparities faced by STs as well as the large degree of variation between tribes. Among 65,941 married women in eight Northeastern states, Mog et al*.* found that 95% of ST women were aware of modern contraceptive methods [17]. However, knowledge may vary significantly by contraception type, with another study demonstrating that while 75.0% of ST women from the state of Chhattisgarh knew about the contraceptive pill, only 16.2% and 7.3% had heard of injectable contraceptives and emergency contraception, respectively [19]. Rates of contraception use were similarly variable in distribution. Use of modern contraceptive methods ranged from 59.2% in a tribal area of Maharashtra, to 48.9% among ST women in Sikkim, to 41.1% among tribal women in West Bengal, to only 11.4% among ST women in Manipur [17, 21, 22]. In a 2014 study, Prusty demonstrated differences between ST women’s modern contraceptive use (39%) and non-tribal women’s modern contraceptive use (47%) across India, with variations in this disparity between several individual states [19]. In the only study in South India, Sreedevi et al*.* reported that 41.2% of ST women had heard of contraceptives, with only a quarter (26.4%) reporting current use of any contraceptive [16].

While national and state-level datasets provide general information on contraception use amongst STs, these findings from specific geographical areas demonstrate that the cultural and socioeconomic heterogeneity of individual tribes may present unique challenges to contraception uptake. Research is therefore needed to explore use of family planning services and contraception needs within distinct tribal groups. We focus on the *Jenu Kurubas*, a tribe who originally resided in state-protected reserve forests of Karnataka, where they pursued a mixed strategy of foraging, wage labor, and wild honey collection, for which they are known [23]. *Jenu Kurubas* are a population of approximately 40,000, with the vast majority living outside of their ancestral lands after the Tiger Reserve Wildlife Act forced evictions of tribal peoples to establish several large tiger preserves in South India [24, 25]. We are currently aware of only one previous study on contraceptive use among the *Jenu Kuruba* tribe that took place in 1998 [26]. By exploring contraceptive knowledge, practices, and sociodemographic determinants of contraceptive awareness among a population of *Jenu Kuruba* women living in Karnataka, we aimed to characterize how *Jenu Kuruba* women’s contraceptive use differs from that of other tribes and to identify gaps that need to be addressed to increase their uptake of contraception. Our results may additionally contribute to the broader body of literature on factors influencing contraceptive use among tribal women in India.

## Methods

### Study sample/population

This cross-sectional survey was conducted between 2011 and 2014 in Mysore District, Karnataka. According to the 2011 census, the population of Mysore District is just under 3 million. More than half (58.6%) of residents live in the district’s 1,332 rural villages [27]. For rural residents, the annual per capita income is INR 16,086 [USD $322] and the literacy rate is 63.3% compared with an all-India annual per capita income of INR 38,005 [USD $760] and literacy rate of 74.0%. The majority of residents are Hindu (88%), 10% are Muslims, and the remainder other religions [27, 28]. Within Karnataka, 11% of women are members of a scheduled tribe [15].

Primary data on contraceptive use and knowledge were collected as part of a larger cohort study called the ‘Saving Children, Improving Lives’ (SCIL) Project conducted by the Public Health Research Institute of India (PHRII). This is a subsample of the SCIL cohort where women who were pregnant, over the age of 18 years, and living in the Mysore subdistrict were invited to participate in a program. This sub study is of a sample of women who belonged to the Jenu Kuruba tribe from the parent study. Detailed description of the SCIL project is provided elsewhere [29]. SCIL was developed to address barriers to HIV-testing among rural women in Mysore District. SCIL provided antenatal care integrated with HIV-testing to women in rural and tribal communities using mobile clinics. Study staff provided health education and community mobilization through the engagement and training of peer health educators. Of the 1948 pregnant women who attended the mobile clinics in SCIL, 303 women self-identified as belonging to *Jenu Kuruba* community. This study reports on those 303 *Jenu Kuruba* women who had recently given birth.

### Measures

#### Outcome variables

The primary outcome variable assessed was knowledge of modern contraception. The variable was dichotomous in nature where we asked each participant if she had ever heard of (*yes/no*) and if she knew where to get (*yes/no*) four temporary forms of birth control (*condoms, injectables, copper-T intrauterine device, and oral contraceptive pill*) and two permanent methods (*female sterilization and male sterilization*). We also collected information on birth control usage, where we asked whether the participant or her partner had ever used (*yes/no*) each of the four contraceptive temporary methods or the two permanent contraceptive methods.

#### Explanatory variables

Sociodemographic data, medical history, obstetric history, knowledge, attitude and beliefs about HIV, and factors influencing institutional delivery were collected using an interviewer-administered questionnaire in *Kannada.* Collected sociodemographic characteristics included participant age (*years*), participant’s and their husband’s education level (*none, 1–8 years, or 9 or more years*), years of marriage, monthly household income *(*< *4,000, 4,001–10,000, or* > *10,000 rupees*), caste or tribe, and number of living children. Other factors assessed included age at first pregnancy, place of last delivery (*home, government facility – sub-center/primary health center/ district health center, private facility – maternity/private nursing home)* and primary decision-maker in the family (*self, participant’s husband, jointly with husband, others in the household, and jointly with others in the household*).

All data were collected by trained interviewers in *Kannada* in paper form and entered in English into Microsoft Access (Redmond, WA) for analysis.

### Statistical analysis

All data were analyzed using StataSE V14.1 (College Station, TX). Descriptive statistics were performed using frequencies and proportions for categorical variables. Bivariate analyses were conducted to determine differences in sociodemographic characteristics between women who reported knowing at least one form of temporary contraception versus those who did not. Multivariable logistic regression analyses were conducted to examine factors associated with contraceptive knowledge. Results of the logistic regression were reported as adjusted odds ratios (AOR) and associated 95% confidence intervals (95%CI).

### Ethical considerations

The study was reviewed and approved by the Institutional Review Boards of the Public Health Research Institute of India (Protocol number: 2011–03-26–10) and Florida International University (Protocol number: IRB-18–0366-AM01), USA. All women provided written informed consent before participating in the program.

## Results

In this study of tribal women in Mysore, India, all participants were married and belonged to the *Jenu Kuruba* (84.5%) or other scheduled tribes living among the *Jenu Kuruba* (15.5%). Table 1 describes the sociodemographic characteristics of this study population. The median age of the women was 28 [IQR 33–23], and 27% reported having no education. Almost half of respondents completed 1–8 years of education (44.8%). Most families (68.0%) earned between 4,000–9,999 Indian Rupees (INR) per month. A majority of women had more than one living child (69.6%). The median age at first pregnancy was 18 [IQR 20–17], with the most common place of last delivery being a government facility, that is either a sub-center, primary health center or district health center (53.3%), or home (41.4%). When asked about household decision-making, most women said that decisions were made solely by her husband (47.2%) or jointly with her husband (24.4%).

**Table 1:** Sociodemographic characteristics of tribal women who have recently delivered in rural Mysore (*N* = 303)

Characteristics	n or Median [IQR]	%
**Age, years**	28 [32–23]	
**Education**		
None	82	27.1
1–8 years	148	48.8
9 + years	73	24.1
**Husband’s Education**		
None	68	22.4
1–8 years	156	51.5
9 + years	79	26.1
**Years of Marriage**	8 [15–4]	
**Monthly household income, Indian Rupees (INR)**		
< 4,000	53	18.9
4,000–9,999	191	68.0
> 10,000	37	13.2
**Caste /Tribe**		
*Jenu Kuruba*	256	84.5
*Betta Kuruba*	11	3.6
*Soliga*	24	7.9
*Yarava*	4	2.3
*Other tribal group*	5	1.7
**Age at first Pregnancy, years**	18 [20–17]	
**Number of living children**		
0	10	3.30
1	85	28.1
2	78	33.3
3	74	21.8
4 or more	70	13.5
**Place of last delivery**		
Home	125	41.4
Sub-center/Primary Health Center/District Health Center	161	53.3
Maternity/Private Nursing Home	16	5.30
**Family decision-maker**		
Self	36	11.9
Husband	143	47.2
Jointly with husband	74	24.4
Others in the household	25	8.3
Jointly with others in the household	25	8.3

Table 2 describes their knowledge of different contraceptive methods and current use of contraception. Overall awareness of temporary methods of contraception was low. Only 39.3% of women reported hearing about one or more forms of temporary contraception, and only 36.3% of those knew where to get them. Of the 303 women surveyed, one-third (100, 33.0%) had heard about copper-T intrauterine device, one-fourth (72, 23.8%) had heard of oral contraception, few (36, 11.9%) had heard of condoms, and very few (14, 4.6%) had heard of injectable contraception. Only seven (2.3%) women reported ever using any form of temporary contraception method.

**Table 2:** Knowledge and use of contraception among tribal women who have recently delivered in rural Mysore (*N* = 303)

Contraceptive Method		Has ever heard of	Knows where to get	Has ever used	Currently using (*n* = 147)
		**n (%)**	**n (%)**	**n (%)**	**n (%)**
Temporary	OCP^a^	72 (23.8)	65 (21.5)	2 (0.7)	0 (0)
	IUD^b^ (Copper-T)	100 (33.0)	90 (29.7)	2 (0.7)	1 (0.7)
	Injectables	14 (4.6)	13 (4.3)	0 (0)	0 (0)
	Condoms	36 (11.9)	34 (11.2)	4(1.3)	2 (1.4)
Permanent	Female Sterilization	271 (89.7)	264 (87.1)	135 (45.0)	114 (77.6)
	Male Sterilization	73 (24.1)	68 (22.5)	2 (0.7)	1 (0.68)

^a^Oral Contraceptive Pill

^b^Intrauterine Device

**Fig. 1:**
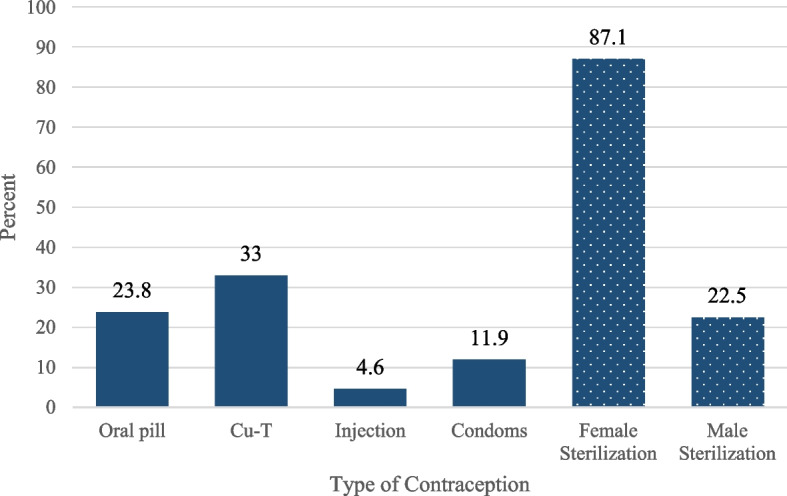
Percentage of women reporting awareness of birth control methods by birth control type (*n* = 303)

**Fig. 2:**
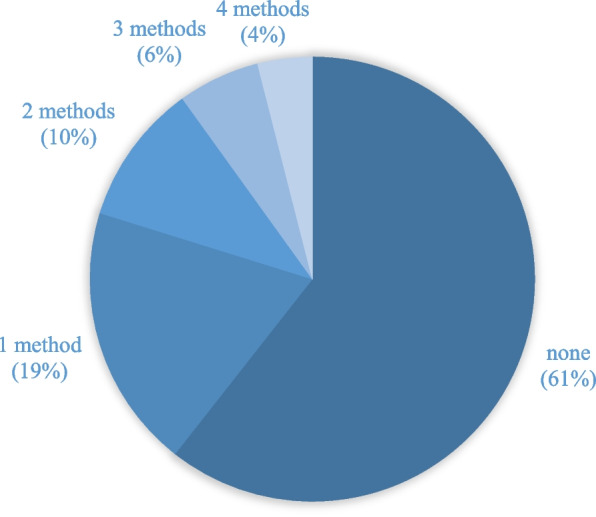
Number of temporary contraceptive methods ever heard of (max = 4) (*n* = 303)

Figure 1 represents the percentage of women who were aware of each birth control type. Most women (87.1%) were aware of female sterilization and the method women were least aware about was injectable contraceptives (4.6%). Figure 2 presents the number of contraceptive methods the women were aware about. Almost two-third (60%) were unaware of any methods and the maximum number of methods women were aware about was four methods (4%).

Table 3 depicts the factors associated with at least one form of contraception through multivariable logistic regression. The results of this analysis indicated that knowledge of at least one form of contraception was linked to age, years with husband, place of last delivery, and household decision-making after adjusting for other variables that were significant (*p* < 0.20) in the univariate analysis. Higher age (AOR: 1.09; 95% CI: 1.02, 1.17) was associated with greater odds of knowing at least one temporary method of contraception, while higher number of years of marriage was associated with lower odds (AOR: 0.90; 95% CI: 0.85, 0.96). Women who reported jointly making household decisions with their husband (AOR: 4.00; 95% CI: 1.41, 11.34) or with other members of the household (AOR: 5.88; 95% CI: 1.63, 21.15) had significantly higher odds of having contraceptive knowledge. Women reporting herself (AOR: 0.52; 95% CI: 0.16, 1.73) or her husband (AOR: 1.40; 95% CI: 0.53, 3.72) as the primary household decision-maker had significantly lower odds of contraceptive knowledge, but this was not statistically significant. Women who had last delivered in a sub-center, primary health center, or district health center (AOR: 3.67; 95% CI: 1.99, 6.82) or a private birthing facility (AOR: 5.88; 95% CI: 1.63, 21.15) had higher odds of having contraceptive knowledge compared to women who delivered at home. Higher education (more than nine years) was associated with increased odds of contraceptive knowledge in the unadjusted model but was not significant in the adjusted model.

**Table 3:** Factors associated with knowledge of at least one form of temporary contraception (*N* = 303)

Characteristics	Unadjusted odds ratio (95% CI)	Adjusted odds ratio (95% CI)
**Age**	**0.95 (0.91, 0.99)**	**1.09 (1.02, 1.17)**
**Years of Marriage**	**0.93 (0.89, 0.97)**	**0.90 (0.85, 0.96)**
**Education**		
No education	REF	REF
1–8 years	1.07 (0.60, 1.90)	0.93 (0.49, 1.79)
9 + years	**2.61 (1.36, 5.01)**	1.64 (0.77, 3.50)
**Husband’s Education**		
No education	REF	–
1–8 years	0.99 (0.55, 1.81)	–
9 + years	1.88 (0.97, 3.66)	–
**Monthly household income, Indian Rupees (INR)**		
< 4,000	REF	–
4,000–9,999	0.60 (0.33, 1.07)	–
> 10,000	1.51 (0.66, 3.46)	–
**Age at first pregnancy**		–
> 18 years	REF	
	1.06 (0.98, 1.15)	
**Number of Living Children**		
0	REF	–
1	2.94 (0.59, 14.67)	–
2	4.42 (0.89, 21.83)	–
3	1.87 (0.36, 9.56)	–
> 4	0.82 (0.14, .4.73)	–
**Place of last delivery**		
Home	REF	REF
Sub-center/Primary Health Center/District Health	**3.13 (1.87, 5.23)**	**3.67 (1.99, 6.82)**
Maternity/Private Nursing Home	**4.07 (1.40, 11.87)**	**4.35 (1.31, 14.44)**
**Household Decision Maker** (REF: No)		
Self	**0.40 (0.18, 0.91)**	0.52 (0.16,1.73)
Husband	**0.53 (0.33, 0.85)**	1.40 (0.53, 3.72)
Jointly with husband	**2.23 (1.31, 3.80)**	**4.00 (1.41,11.34)**
Others in the household	**3.67 (1.53, 8.80)**	**5.88 (1.63, 21.15)**
Jointly with others in the household	0.86 (0.37, 2.01)	–

Multivariable analysis was adjusted for the following independent covariates: age, education, location of last delivery, and household decision maker

## Discussion

Our results showed that knowledge about contraception was generally low among the participants and varied by contraceptive type. We found that few of the ST participants were aware of temporary forms of contraception. About half of women had been sterilized (tubal ligation or laparoscopy), and only about 2% had ever used a temporary method of contraception. This finding is similar to previous studies where a preference for sterilization has been observed in tribal groups in India with possible reasons being that it is a one-time effort, there is a lack of knowledge about temporary methods and the basket of contraceptives offered by the National Family Planning Programme (male and female sterilization, post-partum IUDs, condoms and oral contraceptives) and there are government financial incentives to undergo sterilization [30, 31]. Our study demonstrates the need for education and promotion regarding temporary forms of contraception in this population. Prior studies assessing awareness of contraceptive methods among rural or tribal groups revealed similar low knowledge and use of temporary contraceptive methods [26, 32–34]. Research has also shown that the low adoption of spacing or temporary methods of contraception among tribal groups could be associated with social vulnerabilities such as low literacy and education, younger age at marriage, and lack of privacy in a family setting [35]. In addition, the only previous study we found on the *Jenu Kuruba* community highlighted their preference for indigenous medicines over modern and temporary methods for contraception [26]. The unmet need for family planning specifically in the tribal groups has been previously evidenced before with a key contributor being lack of access to health care services [16, 19, 36]. A study also found that the gap between knowledge and practice of contraceptive methods was wider among tribal than non-tribal groups, due to inadequate and inconsistent interface with the health system [19]. Our finding that women who had given birth in a government facility had more than three times the odds of contraceptive knowledge supports this assertion.

Though a majority of women surveyed had given birth in a government center, a substantial number (41.4%) delivered at home. A policy with significant impacts in this population is the introduction of Janani Suraksha Yojana (JSY). JSY is a way to increase institutional deliveries by providing financial incentives to pregnant women and the community health workers, Accredited Social Health Workers (ASHAs), who accompany the pregnant woman to the health care facility [37]. Having ASHAs to work with this hard-to-reach community will enable the Jenu Kuruba women to approach and navigate the health system and provide opportunities for safer deliveries.

In this study, age was associated with higher odds of contraceptive knowledge while years of marriage was associated with lower odds. That is, older women were more likely to have contraceptive knowledge but women who were married for a longer time were less likely to have contraceptive knowledge. Similar results were found among tribal women of two other Indian states, Jharkhand and Madhya Pradesh [19]. Other research in rural India has shown that women rely on social learning to acquire reproductive health knowledge. Since tribal couples typically follow a nuclear family set-up, the women have less access to other women in the family such as mothers-in-law and grandmothers and thus may be less likely to learn about reproductive health through social learning [38].

Additionally, the multivariable analysis revealed that the household decision maker was a significant predictor of knowledge regarding contraceptive methods. When a woman reported making household decisions jointly with her husband or when others made household decisions, the woman’s odds of contraceptive knowledge increased four or six-fold, respectively. That is, whenever the household decision-making process was shared between a woman and her husband or with others in the household, the woman was found 4–6 times more likely to have knowledge about contraceptives. These odds, however, should be interpreted with caution due to low variation in responses and inflated confidence intervals. Odds of contraceptive knowledge were lower when the woman or her husband made household decisions alone, though this finding was not statistically significant in multivariable regression.

In the present study, income was not a significant predictor of contraception knowledge. Since tribal groups are a notoriously underserved group and the majority of the households studied fall below the poverty line, income was not a good proxy for socioeconomic status. In prior studies in India and worldwide, level of education has been a consistent and significant predictor of contraceptive knowledge and use [39–42]. However, in our adjusted model, education was not significant when controlling for other sociodemographic variables such as age and years of marriage. As with income, education may not play the same role in expanding awareness about reproductive health in this cultural context as it does for the general population. *Jenu Kurubas* in this region reported that despite their levels of education, they continue to face many challenges finding jobs other than daily wage-labor on agricultural farms. Furthermore, although education and income are indicators of upward social mobility, *Jenu Kurubas* are a socially-closed network and tend to only interact with other *Jenu Kurubas* [43].

### Limitations of the study

The results of this study must be interpreted in light of several study limitations. A major limitation was employing a non-probability sampling technique, which could have led to selection bias. In addition, findings from this study cannot be generalized to other populations or tribal groups. As the behaviours and practices were self-reported, there is also a possibility of social desirability bias and recall bias. Data for this study were collected as a cross-sectional survey, and hence we cannot determine any causal associations.

### Strengths of the study

Despite these limitations, there were several strengths to this study. Our study is one of few to study the *Jenu Kuruba* tribal populations among whom we were able to clearly document the lack of knowledge about temporary methods of contraception. There is limited literature on birth spacing methods in peri-urban and rural communities, which is further limited when it comes to indigenous groups and their preferences. We believe that this study contributes to a better and deeper understanding of the gaps in awareness and use of birth spacing or temporary contraception methods among this population of tribal women in South India.

We believe that these findings hold important implications for family planning education and implementation of programs targeting tribal populations in India and suggest targeting young mothers and women delivering at home. We cannot have a one-size-fits-all approach for some of these hard-to-reach communities who often have limited interactions with the health system. We have to promote culturally adaptable interventions that include education and awareness for the entire community and extended family, so that women are supported in their home environment. Furthermore, we need our community and wellness centers to be able to give information about temporary birth spacing methods that are often not adequately discussed or immediately available to these women.

## Conclusion

This study revealed poor knowledge and utilization of temporary contraception methods among a tribal population in rural Mysore, India. We also found a that a significant number of women in this study gave birth at home and those who had institutional deliveries had better knowledge of modern contraceptive methods. Sociodemographic factors such as income and education did not affect contraception knowledge and use. Interventions focused on increasing health care access, contraceptive knowledge and use should be tailored to address the specific needs the tribal population. Policies should be created to capitalize on family education through tribal social networks. These strategies will not only build their trust in the health care system, but will provide opportunities for capacity-building in this under-resourced population.

## Data Availability

The datasets used and/or analyzed during the current study are available from the corresponding author on reasonable request.
